# Clinical and microbiological profile of non-tuberculous mycobacterial endophthalmitis—experience in a tertiary eye care centre in Southern India

**DOI:** 10.1186/s12348-016-0096-x

**Published:** 2016-07-20

**Authors:** Remya Mareen Paulose, Joveeta Joseph, Raja Narayanan, Savitri Sharma

**Affiliations:** Smt. Kanuri Santhamma Centre for Vitreo-Retinal Diseases, L. V. Prasad Eye Institute, Hyderabad, India; Jhaveri Microbiology Centre, Brien Holden Eye Research Centre, L. V. Prasad Eye Institute, Hyderabad, Telangana India

**Keywords:** Infection, Microbiology, Endophthalmitis, Non-tuberculous mycobacteria

## Abstract

**Background:**

Endophthalmitis caused by non-tuberculous mycobacteria (NTM) is a rare condition seen after surgery and trauma. This study reports a retrospective, consecutive, non-comparative case series of 5 patients referred to L. V. Prasad Eye Institute, Hyderabad, and diagnosed with culture-proven NTM endophthalmitis between January 2004 and April 2015. Data collected included demographic information, presenting features, microbiology investigation, treatment course, and final visual outcome.

**Results:**

Of 5555 clinically diagnosed infective endophthalmitis patients, vitreous samples were culture positive for bacteria in 1541 (27.7 %). The isolates from five (0.32 %) patients were identified as NTM. The clinical settings included post-cataract surgery (*n* = 3), post-vitrectomy (*n* = 1), and Descemet’s stripping endothelial keratoplasty (*n* = 1). The species of NTM identified were Mycobacterium chelonae (*n* = 3), Mycobacterium manitobense (*n* = 1), and Mycobacterium fortuitum (*n* = 1). All isolates were sensitive to amikacin while three of the five isolates were sensitive to vancomycin. Initial treatment strategies included pars plana vitrectomy with intravitreal antibiotic (vancomycin and amikacin) injection (*n* = 3), additional intraocular lens explant (*n* = 1), and silicone oil removal in the patient with post-vitreo-retinal surgery. Intravitreal steroid along with antibiotics were given in three patients. Final outcome was favourable (20/200) in one patient, two eyes had unfavourable outcome with multiple recurrences, one was advised evisceration, and one resulted in phthisis bulbi.

**Conclusions:**

This communication reports a series of five cases of NTM endophthalmitis. Poor outcome despite cultureguided therapy suggests virulent nature of the organisms and the need for better treatment strategies.

## Background

Mycobacteria other than *Mycobacterium tuberculosis* are termed as atypical mycobacteria or non-tuberculous mycobacteria (NTM). The first reported ocular infection by NTM was a case of chronic keratitis caused by *Mycobacterium fortuitum*, following the removal of a corneal foreign body in 1965 [1]. Since then, these micro-organisms have been implicated in the aetiology of a variety of ocular infections. NTM are found ubiquitously in the environment in soil, dust, and water [1, 2]. These atypical mycobacteria can be acquired from the natural environment and nosocomially. Infections caused by NTM range from external adnexal infections [3] to keratitis [4–6], scleritis [7], uveitis [8], endophthalmitis [9–11], and panophthalmitis [1]. Risk factors for ocular NTM infections include trauma, previous corneal infection or surgery, corticosteroid use, and systemic immunosuppression [11]. Although NTM are found to be sensitive to the commonly used antibiotics including quinolones and aminoglycosides, their susceptibility may vary [12].

The cases of NTM endophthalmitis reported in the literature [9, 10, 13, 14] have been mostly preceded by an intervention (28/37 eyes, 75.7 %), mainly cataract surgery with intraocular lens (IOL) insertion (18/37 eyes, 48.6 %) [14]. Other predisposing procedures included penetrating keratoplasty, intravitreal injection, scleral buckling, filtering surgery, and Descemet’s stripping automated endothelial keratoplasty or DSAEK (1 eye, 2.7 %) [14, 15]. Herein, we report five cases of culture-proven NTM endophthalmitis. The purpose of this study is to report our experience with the risk factors, microbiological results, clinical findings, response to therapy, and the clinical outcome in patients with this rare disease.

## Methods

This study is a retrospective, consecutive, non-comparative review of medical and microbiology records of all patients diagnosed and treated for NTM endophthalmitis. The patients reported at L. V. Prasad Eye Institute, Hyderabad, India, between January 2004 and April 2015 with postoperative endophthalmitis after having undergone certain ocular surgical procedures elsewhere. The study was approved by institutional review board (LEC-06-15-070) and has been performed in accordance with the ethical standards as laid down in the 1964 Declaration of Helsinki. Data collected included demographic details, cause and duration of symptoms, presenting and final visual acuity, surgical interventions, and microbiology data. All patients had undergone complete eye examination including slit lamp biomicroscopy and indirect ophthalmoscopy. Ultrasound B scan was done in all eyes where the fundus view was hazy. Vitrectomy was done in all cases, and at the time of vitrectomy, all eyes received intravitreal antimicrobial injection with or without corticosteroid. The individual treating physicians made treatment and management decisions. Additional intravitreal injections were given whenever indicated. Vitreous sample was sent immediately to the microbiology laboratory. Explanted IOL and anterior chamber exudate was also sent for culture, wherever indicated.

The smears for microscopy were made from undiluted vitreous/aqueous samples and stained with calcofluor white, Gram, and Giemsa stains. The samples were also inoculated directly onto two 5 % sheep blood agar and one each of 5 % sheep blood chocolate agar, Sabouraud dextrose agar, potato dextrose agar, thioglycollate broth, and brain-heart infusion broth. All media were incubated at 37 °C except the Sabouraud dextrose agar and the potato dextrose agar, which were incubated at 25–27 °C for a period of 7 days. Chocolate agar was incubated in the presence of 5 % CO_2_, and blood agar for anaerobic culture was incubated in anaerobic pouch (Himedia, Mumbai). All other media were incubated aerobically. A culture was considered positive when there was growth of the same organism on two or more media, and/or confluent growth at the site of inoculation on one solid medium, and/or growth in one medium with consistent microscopy findings. Only unequivocal or significant culture results were considered for inclusion in the study. In case of positive cultures, the bacteria were further processed for identification and antibiotic susceptibility testing. All isolates were identified by conventional biochemical tests. Identification of one isolate was confirmed by polymerase chain reaction (PCR)-based 16S ribosomal DNA (rDNA) sequencing [16]. In most cases, treatment was started based on smear report. If response to medication was inadequate, the treatment was modified according to the culture and antibiotic susceptibility report from the laboratory.

## Results

Of 5555 clinically diagnosed infective endophthalmitis patients investigated during the study period, vitreous samples were culture positive for bacteria in 1541 (27.7 %). In five patients (0.32 %), the isolates were identified as NTM. The demographics and clinical setting for each case is summarized in Table 1.

**Table 1:** Clinical profile of five patients with non-tuberculous mycobacterial endophthalmitis

S. no	Age	Sex	Cause/setting	Duration (days)	Clinical findings	Initial BCVA	Primary intervention	IOAB (repeat)	Prognosis	Reason	Final BCVA
1	32	F	ECCE+ PCIOL	300	AC reaction, endoexudates, vitritis grade 2	CF3M	PPV + IOAB (V + C)	NIL	Favourable	–	20/200
2	32	M	BB+ PPV+ EL+SOI	13	AC reaction, endoexudates, vitritis grade 4	PL+	SOR + IOAB (V + C + amp B)	V + C	Unfavourabe	Phthisis	PL-
3	69	F	PHACO + IOL	90	Hypopyon, pas, exudative membrane on IOL, vitritis grade 4	PL+	PPV + IOAB (V + amp B)	V + A + Dx	Unfavourable	Evisceration	PL-
4	50	M	SICS+ PCIOL	29	Hypopyon, vitritis grade 3	CF 1m	PPV + VIT BX + IOAB (V + Vo)	V + A + Dx	Unfavourable	Recurrent vitritis, optic atrophy	PL+
5	64	F	DSEK	45	PAS+, AC reaction, vitritis 3+	PL+	PPV + IOL Explant + IOAB (V + Dx)	V + Dx	Unfavourable	Recurrent vitritis, optic atrophy	PL+

*M* male; *F* female; *HM* hand movements; *LP* light, perception; *SICS* small incision cataract surgery; *ECCE* extra capsular cataract extraction; *PCIOL* posterior chamber intraocular lens; *PPV* pars plana vitrectomy; *SOI* silicone oil injection; *SOR* silicone oil removal; *DSEK* Descemet’s stripping endothelial keratoplasty; *IOAB* intraocular antibiotics; *V* vancomycin; *C* ceftazidime; *A* amikacin; *amp B* amphotericin B; *Dx* dexamethasone; *BCVA* best-corrected visual acuity; *CF* counting fingers; *PL+* accurate projection of light; *PR+* accurate projection of rays

There were three females (60 %) and two males (40 %) in the study group, and the age range was from 32 to 69 years with a mean of 49.4 ± 17.34 years. The time interval between the onset of infection and the initial presentation ranged from 13 to 300 days (mean 95.4 ± 17.34 days). Clinical settings included post-cataract surgery in three patients (uncomplicated phacoemulsification in one and manual small incision cataract surgery in the other and the third one after extracapsular cataract extraction), vitreo-retinal surgery in one patient for rhegmatogenous retinal detachment, and Descemet’s stripping endothelial keratoplasty (DSEK) for pseudophakic bullous keratopathy in one. Initial ultrasound B scans showed low reflective membranous echoes in vitreous with attached retina in all patients with increased choroidal thickness (mean, 1.62 mm).

The major symptoms at presentation were pain, redness, watering, and defective vision. Visual acuity at presentation ranged from light perception in three patients (60 %), counting fingers at 1 m in one (20 %), and counting fingers at 3 m in another (20 %). While the view of the fundus was hazy in patient no. 2, the infection was seen to evolve rapidly and was characterized by a marked anterior chamber reaction, hypopyon, and a severe vitreous humour inflammatory reaction in others. None of these patients had corneal involvement. Four out of five (80 %) patients underwent pars plana vitrectomy with intravitreal antibiotics (vancomycin 1 mg/0.1 ml and amikacin 0.4 mg/0.1 ml or ceftazidime). While one (20 %) patient had intravitreal dexamethasone along with the antibiotic initially, two other patients were given intravitreal dexamethasone during their secondary intervention. Based on clinical suspicion of fungal involvement, two patients were given intravitreal amphotericin B (5 μg/0.1 ml) and one had intravitreal voriconozole. All patients were also started on topical ciprofloxacin, atropine, and prednisolone acetate eye drops. None of the patients were clinically immunocompromised. Additional treatments were tailored according to the response of the eye, and the recurrences were treated appropriately with appropriate antibiotics guided by the sensitivity report.

**Fig. 1:**
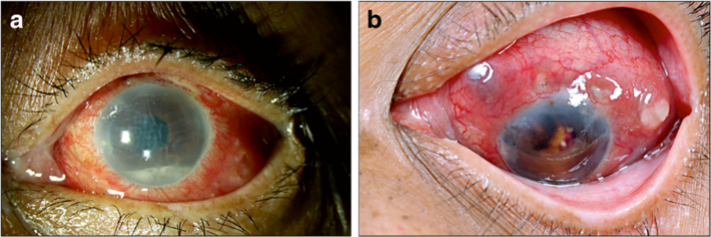
Non-tuberculous mycobacteria endophthalmitis after cataract surgery (patient no. 3). **a** Clinical photograph showing conjunctival congestion, corneal edema, and hypopyon in the anterior chamber. **b** Multiple scleral abscesses at the sites of previous sclerotomy and dark brown exudates in the anterior chamber

Patient no. 3 developed scleral abscesses at the area of the sclerotomies after initial vitreo-retinal procedure (Fig. 1). On final follow-up, she had no light perception with neovascular glaucoma and retinal detachment and was advised evisceration. Of the five patients, only one had a favourable visual outcome (patient no. 1) with late onset endophthalmitis. This patient had presented with visual acuity of counting fingers at 3 m which improved to a final visual acuity of 20/200. Patient no. 2 had phthisis bulbi at the final follow-up, and the other two were quiet after multiple low-grade recurrences, and the final visual acuity was unfavourable in all these cases.

**Fig. 2:**
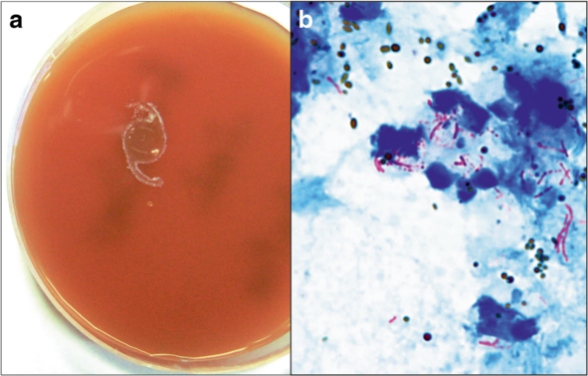
Microbiological investigation of non-tuberculous mycobacteria endophthalmitis—patient no. 5. **a** Growth of tiny, cream, moist bacterial colonies around intraocular lens plated on blood agar (incubation—37 °C, 3 days). **b** Direct smear examination of vitreous lavage showing plenty of polymorphonuclear cells, brown uveal pigments, and pink long, slender, beaded acid fast bacilli (ZN stain, ×1000)

The microbiology findings of the clinical samples are summarized in Table 2. Direct microscopy of smears from vitreous samples was positive in two out of five samples. Based on observation of partially stained beaded bacilli seen in Gram stain, the presence of *Mycobacterium* species was suspected. The smears showed acid fast slender beaded bacilli when restained with Ziehl-Neelsen (ZN) stain (Fig. 2a). In all cases, within 3 days, there was growth of confluent, dry, white colonies on both blood agar (Fig. 2b) and chocolate agar, as well as turbid growth in brain-heart infusion broth in four cases and thioglycollate broth in three cases. Conventional biochemical tests identified three isolates as *Mycobacterium chelonae* and one isolate was *Mycobacterium fortuitum*. The fifth isolate was identified to be *Mycobacterium manitobense* using DNA sequencing. All isolates were sensitive to amikacin, but three isolates were resistant to vancomycin. Four isolates were resistant to ciprofloxacin, and one was resistant to ofloxacin. *M. manitobense*, however, was sensitive to all antibiotics tested.

**Table 2:** Microbiology profile of five patients with non-tuberculous mycobacterial endophthalmitis showing type of species and their antibiotic susceptibility

S. no	Specimen	CFW	Gram	20 % ZN	Culture	No. of days for growth	Organism	A	Cz	Ch	Cip	Gf	Mx	Of	Va	G
1	Vitreous	–	–	–	BA	3	*M. chelonei*	S	R	R	R	ND	ND	R	R	S
					CA											
					BHI											
					Thio											
2	Vitreous	–	–	–	BA	3	*M. chelonei*	S	R	R	S	R	R	R	R	S
					CA											
					BHI											
					Thio											
3	Vitreous	–	–	–	BA	3	*M. chelonei*	S	R	R	S	R	R	R	R	S
					CA											
					BHI											
4	Vitreous	–	GPB 0–5/OIF	AFB—plenty/OIF	BA	3	*M. manitobense* ^a^	S	S	S	S	S	S	S	S	ND
					CA											
5	IOL+ Vitreous + AC exudates	–	GPB—plenty/OIF	AFB—plenty/OIF	BA	3	*M. fortuitum*	S	R	R	S	R	R	R	S	ND
					CA											
					BHI											
					Thio											

*ZN* Ziehl-Neelsen, *GPB* gram-positive bacilli, *AFB* acid fast bacilli, *OIF* oil immersion field, *Neg* negative, *ND* not done, *A* amikacin, *Cz* cefazolin, *Ch* chloramphenicol, *Cip* ciprofloxacin, *Gf* gatifloxacin, *G* gentamicin, *Mx* moxifloxacin, *Of* ofloxacin, *Va* vancomycin, *S* sensitive, *R* resistant, *IOL* intraocular lens, *CFW* calcofluor white stain

^a^The identification was confirmed by 16S rDNA sequencing and phylogenetic analysis

## Discussion

This study demonstrates that NTM endophthalmitis are rare nosocomial infection that can pose both diagnostic and therapeutic challenges. All patients had postoperative endophthalmitis following different types of surgery. None of them were related to trauma. In a recent study of 19 study patients with NTM endophthalmitis, the clinical setting also included post-cataract surgery, post-glaucoma implant, post-intravitreal injection, endogenous endophthalmitis, post-pars plana vitrectomy, and post-scleral buckle exposure [15]. Eyes with implants are reported to be almost six times more likely to end up with loss of vision, in contrast to eyes with a history of a foreign body [13]. Previous reports also suggest that patients usually present within days and up to 35 weeks after initial intervention, with an average of 11.5 weeks [13]. Our series of patients had varied duration of presentation with a minimum of 13 days after vitreo-retinal intervention and maximum of 300 days after cataract surgery. Since atypical mycobacteria can be found in tap and distilled water, contaminated water is likely to be a source of the infection. Some mycobacterial species have resistance to commonly used disinfectants, which makes contamination of certain surgical instruments more likely [17]. It is possible that breach in sterility during surgery led to the infection in the former; however, it is difficult to speculate the mode of infection in the latter.

The threshold for clinical suspicion is likely to be high owing to rarity of the condition and may account for delay in diagnosis. The clinical picture mimics infections caused by other low virulent micro-organisms. Clinically, NTM endophthalmitis may mimic *Propionibacterium acnes* endophthalmitis due to the common presentation of exudative membrane between IOL and posterior capsule [18]. In the present study, the patients (#3, #4) who underwent anterior segment surgeries had intense anterior chamber reaction with fluffy endoexudates suggestive of fungal endophthalmitis. Therefore, these patients were empirically given intravitreal antifungal injection in addition to antibacterial antibiotics.

The vast majority of NTM infections are caused by the Runyon group IV organisms *M. fortuitum* and *M. chelonae*. Other less common species include *Mycobacterium sulzi*, *Mycobacterium flavescens*, *Mycobacterium avium-intracellulare*, *Mycobacterium gordonae*, and *Mycobacterium marinum* [18–21]. We have earlier reported the presence of partially stained or unstained gram-positive bacilli in corneal scraping being a clue for the presence of acid fast organism [22]. A similar clue was seen in the microscopic examination of the Gram stain of the vitreous in two of our patients which led to staining with ZN stain. Mycobacterial infection is generally not suspected in postoperative endophthalmitis, and the poorly staining gram-positive bacilli, as in our patient no. 4, at times could be mistaken as diphtheroids (*Corynebacterium* species). Hence, special stains such as Ziehl-Neelsen stain should be used. However, unlike the common pathogenic mycobacteria, the *M. fortuitum* complex group grows well on routine bacteriologic media such as blood agar and MacConkey agar, as well as on the Lowenstein Jensen medium at temperatures ranging from 25 to 40 °C, in less than 7 days. They form colonies that are smooth, rough, or a mixture of both. *M. fortuitum* complex consists of two species—*M. fortuitum* and *M. chelonae. M. chelonae* has three subspecies—*abscessus*, *chelonae*, and an un-named species (known as *M. chelonae*-like organisms). Mycobacteria in this group grow rapidly, forming non-pigmented colonies in culture at room temperature within 3–5 days instead of the 2–3 weeks required by other mycobacteria [14]. In all the patients in this series, the organism could be identified with in a period of 3–4 days and growth was observed on routine blood agar and chocolate agar media. This indicates that NTM can grow on conventional media and broth, and special media are usually not required. Although the members of Runyon group IV produce positive cultures within 7 days, the infection caused by slow growers is likely to be missed on routine microbiology as culture plates are often discarded after 7 days.

Rapidly growing NTM (RGNTM) endophthalmitis should be considered in the differential diagnosis of refractory anterior chamber and vitreous inflammation, as expedient identification and management is necessary to prevent poor visual outcomes. Standard biochemical testing can identify the causative organism but is limited by the inability to distinguish between RGNTM species reliably, as demonstrated by our recent experience with the isolate of case no. 4 that was identified by Vitek 2 compact system as *Corynebacterium pseudodiphtheriticum*. In Ziehl-Neelsen staining using 20 % H_2_SO_4_ (done routinely in our laboratory for all gram-positive bacilli to rule out *Mycobacterium* sp.) of the culture smear, 40–50 % of the cells showed acid fast staining that led us to subject the isolate at a later stage to 16S rDNA sequencing. The sequence analysis confirmed the isolate to be *M. manitobense*, heretofore unreported from vitreous of patients with endophthalmitis [16]. This experience revalidates our practice of Ziehl-Neelsen staining of all culture isolates of gram-positive bacilli. The recent paper by Shah et al. [15] also used DNA sequencing for the identification of seven subspecies of *M. chelonae/abscessus* in their series. We thus recommend the use of polymerase chain reaction (PCR) in conjunction with 16S rDNA sequencing for reliable differentiation of RGNTM ocular infections. However, cultures remain critical to therapy, as antibiotic susceptibility test results on cultured strains are the best guide to antibiotic selection, given the rapidly rising resistance to antimicrobials within RGNTM strains as evidenced in our study.

A favourable outcome defined as final best-corrected visual acuity of 20/200 or better was found in only one patient, which was similar to the study by Shah et al. [15] wherein the visual outcomes in these patients even after treatment were generally poor. This could be attributed to multiple factors including high virulence of the organism and delayed presentation of the patients. The limitations of the current study include its retrospective design, relatively small number of patients, and use of positive vitreous cultures as the inclusion criteria for the study. Despite these limitations, this study provides important prognostic and antibiotic resistance data for endophthalmitis caused by non-tubercular mycobacteria species.

## Conclusions

Based on our study, we may conclude that NTM is an uncommon condition but should be considered in the differential diagnosis of delayed-onset, chronic endophthalmitis after ocular surgeries especially with an implant. Therefore, when rapidly growing mycobacteria are recovered from patients with endophthalmitis, amikacin should be included in the therapeutic regimen until speciation and in vitro susceptibility testing suggest equally efficacious alternatives. Despite the use of sensitive antibiotics, several patients of NTM infection do not respond to medical treatment and require surgical intervention.
